# The Effects of Foot Reflexology on Vital Signs: A Meta-Analysis of Randomized Controlled Trials

**DOI:** 10.1155/2022/4182420

**Published:** 2022-09-13

**Authors:** Yunyan Jing, Shanxin Liu, Chunqi Pan, Ying Jian, Mingwei Wang, Bin Ni

**Affiliations:** ^1^Intensive Care Unit, Hangzhou Normal University Affiliated Hospital, No. 126 Wenzhou Road, Hangzhou, Zhejiang 310000, China; ^2^Department of Cardiology, Hangzhou Normal University Affiliated Hospital, No. 126 Wenzhou Road, Hangzhou, Zhejiang 310000, China

## Abstract

**Introduction:**

We evaluated the effects of foot reflexology on bodily vital signs.

**Methods:**

Randomized controlled trials (RCTs) evaluating the effects of foot reflexology on vital signs were collected for a meta-analysis. Statistical analysis was conducted using RevMan5.4 software and pooled estimates of the effects were reported as mean differences (MDs) with 95% conﬁdence intervals (CIs).

**Results:**

Thirteen studies, including 819 patients, met our inclusion criteria. Our results showed that systolic blood pressure (SBP) (MD = -4.62, 95% CI: -5.58 to -3.66; *P* < 0.00001), diastolic blood pressure (DBP) (MD = -3.32, 95% CI: -4.48 to -2.17; *P* < 0.00001), heart rate (HR) (MD = -4.76, 95% CI: -6.49 to -3.04; *P* < 0.00001), respiratory rate (RR) (MD = -0.77, 95% CI: -1.50 to -0.48; *P* < 0.00001), and pulse oxygen saturation (SpO_2_) (MD = 0.95, 95% CI: 0.39 to 1.52; *P* = 0.0009) showed statistical significance in the foot reflexology group.

**Conclusions:**

Short-term followup results showed that foot reflexology exerted positive effects on vital signs, reduced BP, HR, and RR and increased SpO_2_.

## 1. Introduction

As our society ages, the incidence of cardiovascular diseases is gradually increasing and is accompanied by additional complications that become burdens to families and society. Changes in vital signs, such as blood pressure (BP), heart rate (HR), respiratory rate (RR), and pulse oxygen saturation (SpO_2_) can indicate the state of physiological function and also serve as early warning systems for disease progression. Hypertension and rapid HR are associated with an increased risk of cardiovascular events, increased RR often precedes changes in other vital signs, and for SpO_2_, the dangers of hypoxemia have long been acknowledged [1]. Understanding the significance of changes in vital signs can provide additional clinical help to better predict disease progression and prognosis [2]. In addition to physiological factors, psychological factors also influence changes in vital signs. Negative psychological health encompasses chronic stress, anxiety, depression, and anger [3]. Chronic daily life stress and anxiety affect cardiovascular disease (CVD) health, and CVD development such as myocardial infarction, heart failure, stroke, or coronary revascularization may also lead to the development of negative psychological health [4,5]. Thus, these negative aspects of psychological health must be reduced and physical and mental health promoted. Thanks to the continuous efforts of researchers, the effectiveness of pharmacological treatments for hypertension, CVD, and mental illness are indubitable. However, because of poor compliance and side effects of drugs, many patients' vital signs remain outside normal ranges. This means that drugs cannot completely solve a patient's physical and mental illness, therefore innovative approaches are required to reduce the burden of cardiopulmonary disease [6].

Foot reflexology is a noninvasive and complementary therapy; it comforts and relaxes patients to help them adapt to new situations [7]. The technique is well-known in many countries such as China, India, and Thailand. Several randomized controlled trials (RCTs) have reported that foot reflexology influences BP, HR, RR, and SpO_2_, but with conflicting results [8–20]. Given relatively small sample sizes and these conflicting results, comprehensive conclusions have not been identified. Therefore, to address this, we conducted a meta-analysis on RCTs to determine the effects of foot reflexology on vital signs in patients with cardiovascular disease.

## 2. Methods

This meta-analysis followed the Preferred Reporting Items for Systematic Reviews and Meta-Analyses (PRISMA) 2020 statement [21]. The PRISMA checklist was provided in Supplementary File 1.

### 2.1. Search Strategy

From database inception to 31^st^ July 2022, a systematic literature search was performed using Web of Science, the Cochrane Library, PubMed, Clinicaltrials.gov, and Medline to evaluate the effects of foot reflexology on vital signs. The search was restricted to studies published in English. Search keywords were: “foot reflexology and (blood pressure or heart rate or respiratory rate or SpO2).” We conducted a “snowball search,” where we manually searched the bibliographies of selected studies, reviews, and previous meta-analyses. No articles meeting inclusion criteria were found. A detailed PubMed search strategy is provided in Supplementary File 2.

### 2.2. Study Selection

Selected RCTs satisfied the following inclusion criteria: (I) RCTs comparing vital sign effects in foot reflexology groups when compared with control groups; patients were not limited by gender and age; (II) studies provided data on BP or HR or RR or SpO_2_; and (III) studies published in English. Exclusion criteria: (I) duplicate studies; (II) studies where data could not be extracted; and (III) studies performing reflexology on the foot and other areas such as the hands or back.

### 2.3. Data Collection and Quality Assessment

The following information was extracted from eligible studies: first author, publication year, number of participants in foot reflexology and control groups, followup duration, and main outcomes (BP, HR, RR, and SpO_2_). The Cochrane risk of bias tool was used to assess bias risk in selected studies. A risk of bias graph and a summary of selected studies were both generated using the risk of bias assessment tool for randomized studies (RoBARs tool): (I) randomization process; (II) allocation concealment; (III) blind method; (IV) outcome assessors; (V) missing outcome data processing; (VI) selection of the reported result; and (VII) other bias.

### 2.4. Statistical Analysis

Statistical analysis was conducted using RevMan5.4 software. Continuous variables were expressed as the mean ± standard deviation and mean differences (MDs) with 95% confidence intervals (CIs). Heterogeneity levels in selected RCTs were assessed using I^2^-statistic. A fixed-effect model was used to calculate summary estimates and their 95% CIs if heterogeneity among RCTs was low (I^2^ < 50%); a random-effect model was used if heterogeneity was significant (50% I^2^ < 70%); and RCTs were not be combined if heterogeneity was very significant (I^2^ > 70%) [22]. Sensitivity analyses were performed by removing each study individually to estimate the quality and consistency of results. To assess publication bias, a scatter funnel plot was used to plot risk ratios from selected RCTs on the *X*-axis versus the standard error of the log risk ratio of each RCT on the *Y*-axis [23]. *P* <  0.05 was considered statistically significant. Two researchers independently performed literature searches, examined all titles and abstracts for eligibility, and assessed full-text eligibility after full-texts were obtained. Any divergence was resolved by consensus.

## 3. Results

### 3.1. Study Selection

**Figure 1:**
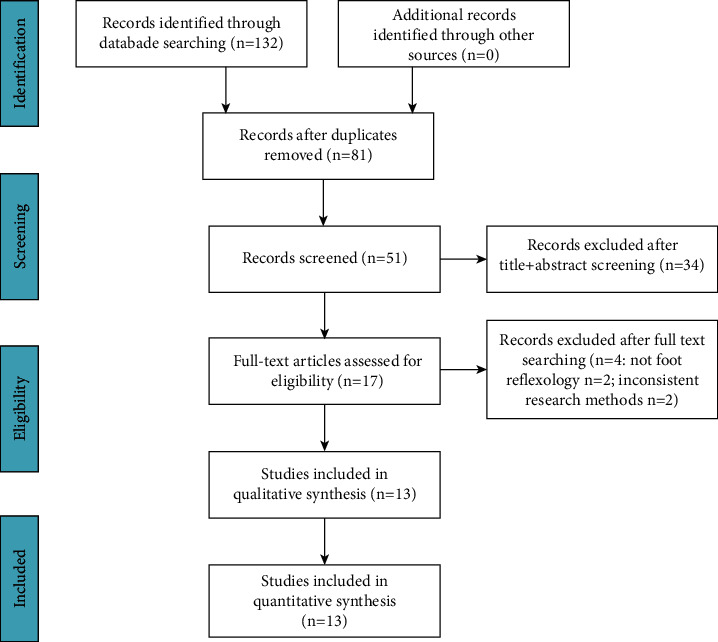
Flow diagram showing literature searches and study selection for analysis.

The initial search produced 132 relevant articles, of which 17 were considered potentially eligible. However, after further reading of abstracts and full texts, four were excluded: two were not related to foot reflexology [24, 25], and the other two were not consistent with the rest of the methodology [26, 27] (Figure 1). Ultimately, we included 13 RCTs.

### 3.2. Characteristics of Included Studies

The 13 RCTs included 819 patients. Of these, 410 patients were randomized to a foot reflexology group, whereas 409 were randomized to a control group. Information on eligible studies is summarized (Table 1).

### 3.3. Quality Assessment

**Figure 2:**
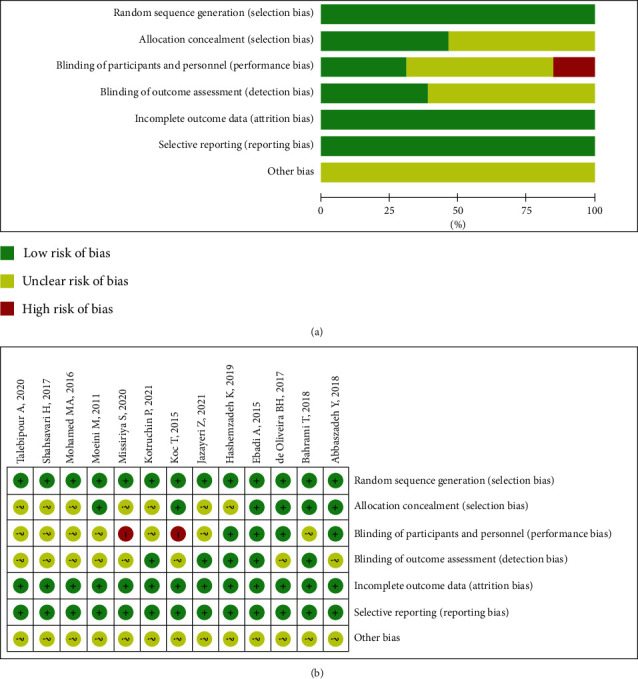
Risk of bias graph and a summary of selected studies using the risk of bias assessment tool for randomized studies (RoBARs) tool. (a) RoBARs graph; (b) RoBARs summary.

For quality assessment, in terms of random sequence generation, incomplete outcome data and selective reporting in all studies demonstrated a low risk of bias. Two studies [11, 18] (<25%) had a high risk of bias in terms of blinding participants and personnel. We observed that >50% of studies had an unclear risk of bias for allocation concealment, blinding of participants and personnel, and blinding of outcome assessment (Figure 2).

### 3.4. Meta-Analysis

Two researchers independently conducted the study with consistent results.

#### 3.4.1. SBP and DBP

**Figure 3:**
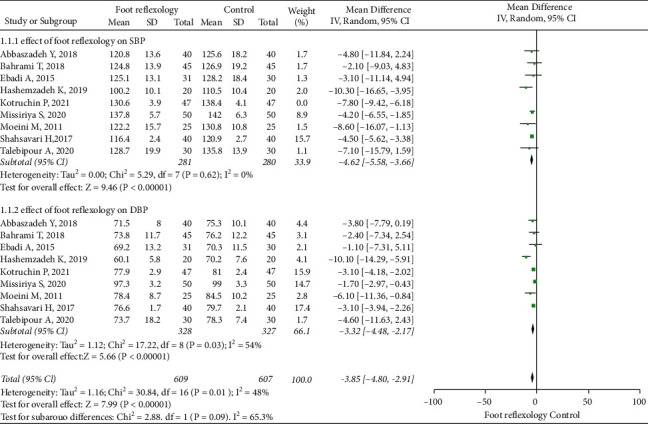
Forest plot showing foot reflexology therapy effects on blood pressure.

Among the 13 RCTs, 8, containing 581 patients, reported information on SBP. Due to large heterogeneity, sensitivity analysis, performed by the exclusion method, showed that one RCT by Kotruchin et al. was the main cause of heterogeneity, therefore we excluded this RCT [8]. Nine studies, with 655 patients, reported information on DBP; sensitivity analysis showed no significant changes in heterogeneity. A random-effect model was applied, with pooled analyses showing SBP (I2 = 0, MD = -4.62, 95% CI: -5.58 to -3.66; Figure 3) and DBP (I2 = 54, MD = −3.32, 95% CI: −4.48 to −2.17; Figure 3) and *P* <  0.05 for all results. Thus, SBP and DBP were statistically significant in the foot reflexology group.

#### 3.4.2. HR And SpO_2_

**Figure 4:**
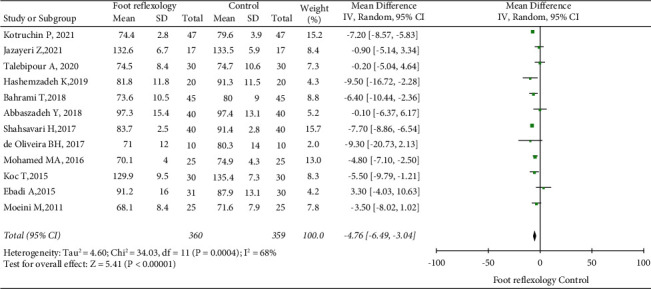
Forest plot showing foot reflexology therapy effects on heart rate; data pooled from 12 RCTs.

**Figure 5:**
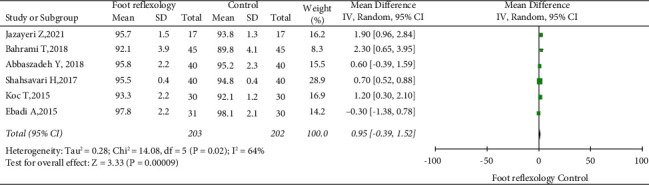
Forest plot showing foot reflexology therapy effects on oxygen saturation; data pooled from six RCTs.

Among the 13 RCTs, 12, with 719 patients, reported information on HR, while 6, with 405 patients, reported information on SpO_2_. Random-effect models were used due to significant heterogeneity among RCTs (50% I^2^ < 70%). Sensitivity analyses showed no significant changes in heterogeneity were observed. We showed that HR (MD = −4.76, 95% CI: −6.49 to −3.04; Figure 4) and SpO_2_ (MD = 0.95, 95% CI: 0.39 to 1.52; Figure 5) were statistically significant in the foot reflexology group, with *P* <  0.05 for all results. Our meta-analysis clearly showed that foot reflexology therapy reduced HR and increased SpO_2_.

#### 3.4.3. RR

**Figure 6:**
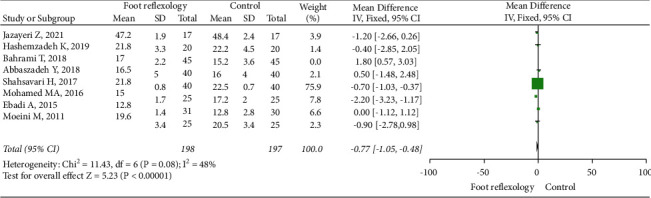
Forest plot showing foot reflexology therapy effects on respiratory rate; data pooled from seven RCTs.

Among the 13 RCTs, 7, with 395 patients, reported information on SBP. RR could not be combined as heterogeneity among studies was significant (I^2^ > 70%). Sensitivity analysis showed that one RCT by Bahrami et al. was the main cause of heterogeneity. After excluding this [13], fixed-effect models indicated that RR decreased in the foot reflexology group (I^2^ = 48, MD = −0.77, 95% CI: −1.50 to −0.48; Figure 6), and showed that foot reflexology therapy reduced RR.

### 3.5. Publication Bias

**Figure 7:**
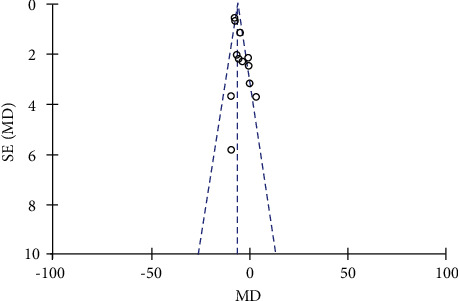
Scatter funnel plot of risk ratios on the *X*-axis against the standard error of log risk ratio on the *Y*-axis.

A funnel plot of studies appeared symmetrical, indicating the absence of publication bias (Figure 7).

## 4. Discussion

Vital signs, such as BP, HR, RR, and SpO_2_ are important clinical status indicators in patients. Changes in vital signs can indicate the state of physiological function and can also serve as an early warning system for disease. Hypertension is a major cause of cardiovascular disease and deaths worldwide, especially in low- and middle-income countries. Despite the availability of therapies, < 14% of adults with hypertension have controlled BP [28]. Clinical trials have shown that maintaining normal BP in hypertensive patients is effective for preventing CVD mortality [29]. Also, when compared with individuals with normal HR, the risk of persistent hypertension in those with a fast HR increases significantly [30]. Pulse oximetry is a noninvasive measurement technique which assesses oxygenation levels and reflects a balance between oxygen delivery and consumption. Increased RR and decreased SpO_2_ indicate the body may be in a state of hypoxia or imbalanced oxygen supply and demand. Therefore, vital signs such as BP, HR, RR, and SpO_2_ are essential for monitoring adverse events [1, 31]. Furthermore, chronic stress and high anxiety levels are associated with altered physiological states, especially in the autonomic nervous system, which can lead to high HR, hypertension, and CVD [32, 33]. Several RCTs have reported that foot reflexology affects cardiovascular-related parameters and reduces patient fatigue, but the results are inconsistent [8–20].

### 4.1. Main Study Results And Issues

Our meta-analysis clearly showed that foot reflexology therapy had a positive effect on adverse changes to vital signs. Foot reflexology therapy reduced BP, HR, RR, and increased SpO_2_. However, many factors may have influenced our results, therefore we analyzed our conclusions from multiple aspects. Our study had several strengths: (I) all studies were RCTs; (2) for quality assessment, in terms of random sequence generation, incomplete outcome data and selective reporting in all studies demonstrated a low risk of bias.

However, although RCTs were of high quality, our study had some weaknesses: (I) subjects in all age groups, from infants to the elderly, including patients and healthy subjects, resulted in small sample sizes in every RCT. (II) Followup times in RCTs were inconsistent and relatively short; (III) subjects were from different countries and races, and foot reflexology manipulations may have been different. Therefore, our final conclusions must be considered with caution. In future research, more detailed criteria are required, such as patients in the same age group, patients with similar diseases, patients of the same race, and more subjects to study.

### 4.2. Possible Mechanisms of Action

A reflex zone corresponds to an organ, gland or body part, and massage pressure on this reflex zone increases blood supply to the corresponding organ. Foot reflexology is an ancient practice where the thumb and fingers are used on the feet to stimulate some reflex zones; the technique promotes well-being, reduces fatigue by increasing vagal modulation, and decreases sympathetic modulation which helps manage adverse physical issues [13, 34]. Currently, there is a limited understanding on how reﬂexology works; however, the following theories may provide some insights. The first theory suggested foot reflexology may function by stimulating the nervous system [35, 36]. Rollinson et al. had similar theories [37] and hypothesized that individual areas on the plantar surface of the feet were linked to distinct bundles of discreet nerve endings. Each individual point was believed to map or correspond to a speciﬁc internal organ within the body.

The second theory posited that reflexology contact points were similar to meridians or channels of energy, on which acupuncture, acupressure, and Shiatsu were based. Unblocking these energy lines or meridians was believed to balance the body [38].

The third theory postulated that foot stimulation activated the parasympathetic nervous system and triggered the release of endogenous chemicals. This theory showed that local skin temperature changes by skin-to-skin contact and local enzymatic reactions in receptive fields improved blood supply and physical function [39]. Thus, many foot reflexology mechanisms may exist but are not described. Regardless of the inherent mechanisms, the true effects of foot reflexology cannot be underestimated.

### 4.3. Similarities And Differences with Other Published Studies

In recent years, several meta-analyses on foot reflexology have been published [40–44] and showed the technique was an effective complementary therapy for treating functional constipation, relieving fatigue, improving sleep disturbance, and improving glycemic control and diabetic peripheral neuropathy. However, no meta-analysis has shown the effects of foot reflexology on vital signs. Although there is a systematic review [45] showing favorable effects of foot therapy on vital signs in terms of BP and cardiac index, only three studies were included in the article and the overall study quality was low, so the effects of foot therapy on vital signs remain unclear and it is not clear how reflexology affects physiological and biochemical parameters. Song et al. [46] conducted a systematic review of three non-RCTs and showed that self-administered foot reflexology significantly improved subjective outcomes such as perceived stress, fatigue, and depression, but no significant improvements in objective outcomes such as BP and HR were found. With increased emphasis and research on foot reflexology, this meta-analysis is the first to include current RCTs to evaluate the effects of foot reflexology on vital signs.

### 4.4. Limitations

Our meta-analysis had some limitations. Firstly, there may have been differences in the manipulation of foot reflexology approaches within RCTs, intervention durations were somewhat different, and variations existed within studies in terms of patient type, which may have caused some bias. Secondly, no long-term follow-up was performed: our results mainly reflected short-term outcomes where the longest followup time was 8 weeks, therefore, the long-term efficacy of foot reflexology therapy for BP, HR, and SpO_2_ requires further study. Thirdly, our sample sizes were small. Finally, our protocol was not preregistered in registration platforms. No relevant studies with negative results were found on registry platforms such as PROSPERO, which may lead to publication bias, although we did not change our plans halfway through and there was no selective reporting bias.

## 5. Conclusions

This is the first RCT meta-analysis evaluating the effects of foot reflexology on vital signs. We showed the technique exerted positive effects on these signs; it reduced BP, HR, and RR and increased SpO_2_. However, our results mainly reflected short-term followup outcomes, therefore, the long-term efficacy of the technique on vital signs must be investigated in future studies.

## Figures and Tables

**Table 1:** Information extracted from selected studies.

First author, publication year	Followup	Patients F/C	SBP (mmHg) F/C	DBP (mmHg) F/C	HR (bpm) F/C	RR (bpm) F/C	SpO_2_ (%) F/C	Quality evaluation
Kotruchin et al.,[8]	30 min	47/47	130.6 ± 3.9/138.4 ± 4.1	77.9 ± 2.9/81 ± 2.4	72.4 ± 2.8/79.6 ± 3.9	—	—	①③④⑤
Jazayeri et al.,[9]	15 min	17/17	—	—	132.6 ± 6.7/133.5 ± 5.9	47.2 ± 1.9/48.4 ± 2.4	95.7 ± 1.5/93.8 ± 1.3	①③④⑤
Talebipour et al.,[10]	30 min	30/30	128.7 ± 19.9/135.8 ± 13.9	73.7 ± 18.2/78.3 ± 7.4	74.5 ± 8.4/74.7 ± 10.6	—	—	①④⑤
Missiriya et al.,[11]	6W	50/50	137.8 ± 5.7/142.0 ± 6.3	97.3 ± 3.2/99.0 ± 3.3	—	—	—	①④⑤
Hashemzadeh et al, [12]	40 min	20/20	100.2 ± 10.1/110.5 ± 10.4	60.1 ± 5.8/70.2 ± 7.6	81.8 ± 11.8/91.3 ± 11.5	21.8 ± 3.3/22.2 ± 4.5	—	①③④⑤
Bahrami T, 2018 [13]	20 min	45/45	124.8 ± 13.9/126.9 ± 19.2	73.8 ± 11.7/76.2 ± 12.2	73.6 ± 10.5/80.0 ± 9.0	17.0 ± 2.2/15.2 ± 3.6	92.1 ± 3.9/89.8 ± 4.1	①②③④⑤
Abbaszadeh et al.,[14]	10 min	40/40	120.8 ± 13.6/125.6 ± 18.2	71.5 ± 8.0/75.3 ± 10.1	97.3 ± 15.4/97.4 ± 13.1	16.5 ± 5.0/16.0 ± 4.0	95.8 ± 2.2/95.2 ± 2.3	①②③④⑤
Shahsavari Het al.,[15]	30 min	40/40	116.4 ± 2.4/120.9 ± 2.7	76.6 ± 1.7/79.7 ± 2.1	83.7 ± 2.5/91.4 ± 2.8	21.8 ± 0.8/22.5 ± 0.7	95.5 ± 0.4/94.8 ± 0.4	①④⑤
de oliveira et al.,[16]	5W	10/10	—	—	71.0 ± 12/80.3 ± 14.0	—	—	①②③④⑤
Mohamed et al.,[17]	8W	25/25	—	—	70.1 ± 4.0/74.9 ± 4.3	15.0 ± 1.7/17.2 ± 2.0	—	①④⑤
Koc and Gozen [18]	30 min	30/30	—	—	129.9 ± 9.5/135.4 ± 7.3	—	93.3 ± 2.2/92.1 ± 1.2	①②④⑤
Ebadi et al.,[19]	20 min	31/30	125.1 ± 13.1/128.2 ± 18.4	69.2 ± 13.2/70.3 ± 11.5	91.2 ± 16.0/87.9 ± 13.1	12.8 ± 1.4/12.8 ± 2.8	97.8 ± 2.2/98.1 ± 2.1	①②③④⑤
Moeini et al.,[20]	30 min	25/25	122.2 ± 15.7/130.8 ± 10.8	78.4 ± 8.7/84.5 ± 10.2	68.1 ± 8.4/71.6 ± 7.9	19.6 ± 3.4/20.5 ± 3.4	—	①②④⑤

SBP: systolic blood pressure; DBP: diastolic blood pressure; HR: heart rate; RR: respiratory rate; SpO2: oxygen saturation; F/C: foot reflexology group/control group; min: minute; W: week; ①: Random sequence generation; ②: Allocation concealment; ③: Blinding of participants and personnel; ④: Incomplete outcome data; ⑤: Selective reporting.

## Data Availability

The data used to support the findings of this study are available from the corresponding author upon request.

## Supplementary Materials

Supplementary Materials Table S. PRISMA 2020 checklist (PRISMA for systematic review and meta-analyses).
