# Supplementary material for: The Effects of Foot Reflexology on Vital Signs: A Meta-Analysis of Randomized Controlled Trials
Source: Evid Based Complement Alternat Med. 2022 Sep 13;2022:4182420. doi: 10.1155/2022/4182420 (PMC9489354; doi:10.1155/2022/4182420)
Supplement: Supplementary Materials — Table S. PRISMA 2020 checklist (PRISMA for systematic review and meta-analyses). [file 4182420.f1.zip › 4182420.f1/Supplementary file 2-search strategy.docx]

The full electronic search strategy for Pubmed:

Search keywords: foot reflexology and (blood pressure or heart rate or respiratory rate or SpO2);

Years searched: From database inception to 31st July 2022.

Search results: The initial search produced 51 relevant articles, 3 were after 31st December 2021, but did not meet inclusion criteria.

In addition, systematic literature searches were performed using Web of Science, the Cochrane Library, Clinicaltrials.gov, and Medline, from 31st December 2021 to 31st July 2022. No articles meeting our inclusion criteria were found.

Search results in Pubmed:

1: Kotruchin P, Imoun S, Mitsungnern T, Aountrai P, Domthaisong M, Kario K. The effects of foot reflexology on blood pressure and heart rate: A randomized clinical trial in stage-2 hypertensive patients. J Clin Hypertens (Greenwich). 2021 Mar;23(3):680-686. doi: 10.1111/jch.14103. Epub 2020 Nov 15. PMID:33190420; PMCID: PMC8029572.

2: Baljon KJ, Romli MH, Ismail AH, Khuan L, Chew BH. Effectiveness of breathing exercises, foot reflexology and back massage (BRM) on labour pain, anxiety, duration, satisfaction, stress hormones and newborn outcomes among primigravidae during the first stage of labour in Saudi Arabia: a study protocol for a randomised controlled trial. BMJ Open. 2020 Jun 15;10(6):e033844. doi: 10.1136/bmjopen-2019-033844. PMID: 32540887; PMCID: PMC7299053.

3: Karatas N, Dalgic AI. Effects of reflexology on child health: A systematic review. Complement Ther Med. 2020 May;50:102364. doi:10.1016/j.ctim.2020.102364. Epub 2020 Mar 3. PMID: 32444044.

4: Zimpel SA, Torloni MR, Porfírio GJ, Flumignan RL, da Silva EM. Complementary and alternative therapies for post-caesarean pain. Cochrane Database Syst Rev. 2020 Sep 1;9:CD011216. doi: 10.1002/14651858.CD011216.pub2. PMID: 32871021.

5: Silva NCM, Chaves ÉCL, Carvalho EC, Carvalho LC, Iunes DH. Effect of Foot Reflexology on Capillary Blood Glucose, Tissue Temperature, and Plantar Pressure of Individuals With Diabetes Mellitus (Type 2): A Pilot Study. J Chiropr Med. 2018 Sep;17(3):182-189. doi: 10.1016/j.jcm.2018.03.003. Epub 2018 Aug 26. PMID: 30228809; PMCID: PMC6141416.

6: Arslan G, Ceyhan Ö, Mollaoğlu M. The influence of foot and back massage on blood pressure and sleep quality in females with essential hypertension: a randomized controlled study. J Hum Hypertens. 2021 Jul;35(7):627-637. doi: 10.1038/s41371-020-0371-z. Epub 2020 Jul 16. PMID: 32678299.

7: Jazayeri Z, Sajadi M, Dalvand H, Zolfaghari M. Comparison of the effect of foot reflexology and body massage on physiological indicators and bilirubin levels in neonates under phototherapy. Complement Ther Med. 2021 Jun;59:102684. doi: 10.1016/j.ctim.2021.102684. Epub 2021 Feb 17. PMID: 33609634.

8: Yilmaz D, Yilmaz Kurt F. The effect of foot reflexology on procedural pain before heel lancing in neonates. Arch Pediatr. 2021 May;28(4):278-284. doi: 10.1016/j.arcped.2021.02.015. Epub 2021 Mar 11. PMID: 33715931.

9: Ghaljaei F, Jalalodini A. The effects of foot reflexology on pain and physiological indicators in children with leukemia under chemotherapy: a clinical trial study. Rep Pract Oncol Radiother. 2021 Dec 30;26(6):955-961. doi: 10.5603/RPOR.a2021.0116. PMID: 34992868; PMCID: PMC8726439.

10: Ejindu A. The effects of foot and facial massage on sleep induction, blood pressure, pulse and respiratory rate: crossover pilot study. Complement Ther Clin Pract. 2007 Nov;13(4):266-75. doi: 10.1016/j.ctcp.2007.03.008. Epub 2007 May 8. PMID: 17950182.

11: Song HJ, Choi SM, Seo HJ, Lee H, Son H, Lee S. Self-administered foot reflexology for the management of chronic health conditions: a systematic review. J Altern Complement Med. 2015 Feb;21(2):69-76. doi: 10.1089/acm.2014.0166. Epub 2015 Jan 30. PMID: 25636044.

12: Song HJ, Son H, Seo HJ, Lee H, Choi SM, Lee S. Effect of self-administered foot reflexology for symptom management in healthy persons: a systematic review and meta-analysis. Complement Ther Med. 2015 Feb;23(1):79-89. doi: 10.1016/j.ctim.2014.11.005. Epub 2014 Dec 9. PMID: 25637156.

13: Lu WA, Chen GY, Kuo CD. Foot reflexology can increase vagal modulation, decrease sympathetic modulation, and lower blood pressure in healthy subjects and patients with coronary artery disease. Altern Ther Health Med. 2011 Jul- Aug;17(4):8-14. PMID: 22314629.

14: Jones J, Thomson P, Irvine K, Leslie SJ. Is there a specific hemodynamic effect in reflexology? A systematic review of randomized controlled trials. J Altern Complement Med. 2013 Apr;19(4):319-28. doi: 10.1089/acm.2011.0854. Epub 2012 Oct 16. PMID: 23072265.

15: Chatchawan U, Jarasrungsichol K, Yamauchi J. Immediate Effects of Self-Thai Foot Massage on Skin Blood Flow, Skin Temperature, and Range of Motion of the Foot and Ankle in Type 2 Diabetic Patients. J Altern Complement Med. 2020 Jun;26(6):491-500. doi: 10.1089/acm.2019.0328. Epub 2020 Apr 28. PMID: 32349513.

16: Tanju O, Fatma Yilmaz K. Effect of acupressure on procedural pain before heel lancing in neonates. J Tradit Chin Med. 2021 Apr;41(2):331-337. PMID: 33825415.

17: Abbaszadeh Y, Allahbakhshian A, Seyyedrasooli A, Sarbakhsh P, Goljarian S, Safaei N. Effects of foot reflexology on anxiety and physiological parameters in patients undergoing coronary artery bypass graft surgery: A clinical trial. Complement Ther Clin Pract. 2018 May;31:220-228. doi: 10.1016/j.ctcp.2018.02.018. Epub 2018 Mar 3. PMID: 29705459.

18: Cicek SC, Demir S, Yilmaz D, Yildiz S. Effect of reflexology on ankle brachial index, diabetic peripheral neuropathy, and glycemic control in older adults with diabetes: A randomized controlled trial. Complement Ther Clin Pract. 2021 Aug;44:101437. doi: 10.1016/j.ctcp.2021.101437. Epub 2021 Jul 4. PMID: 34237668.

19: Alimohammad HS, Ghasemi Z, Shahriar S, Morteza S, Arsalan K. Effect of hand and foot surface stroke massage on anxiety and vital signs in patients with acute coronary syndrome: A randomized clinical trial. Complement Ther Clin Pract. 2018 May;31:126-131. doi: 10.1016/j.ctcp.2018.01.012. Epub 2018 Feb 13. PMID: 29705444.

20: Farmahini Farahani M, Noruzi Zamenjani M, Nasiri M, Shamsikhani S, Purfarzad Z, Harorani M. Effects of Extremity Massage on Preoperative Anxiety: A Three-Arm Randomized Controlled Clinical Trial on Phacoemulsification Candidates. J Perianesth Nurs. 2020 Jun;35(3):277-282. doi: 10.1016/j.jopan.2019.10.010. Epub 2020 Feb 10. PMID: 32057627.

21: Jang SH, Kim KH. [Effects of self-foot reflexology on stress, fatigue and blood circulation in premenopausal middle-aged women]. J Korean Acad Nurs. 2009 Oct;39(5):662-72. Korean. doi: 10.4040/jkan.2009.39.5.662. PMID: 19901496.

22: Eguchi E, Funakubo N, Tomooka K, Ohira T, Ogino K, Tanigawa T. The Effects of Aroma Foot Massage on Blood Pressure and Anxiety in Japanese Community Dwelling Men and Women: A Crossover Randomized Controlled Trial. PLoS One. 2016 Mar 24;11(3):e0151712. doi: 10.1371/journal.pone.0151712. PMID: 27010201; PMCID: PMC4807074.

23: Xue M, Fan L, Ge LN, Zhang Y, Ge JL, Gu J, Wang Y, Chen Y. Postoperative Foot Massage for Patients after Caesarean Delivery. Z Geburtshilfe Neonatol. 2016 Aug;220(4):173-8. doi: 10.1055/s-0042-104802. Epub 2016 Aug 10. PMID: 27509141.

24: Ebadi A, Kavei P, Moradian ST, Saeid Y. The effect of foot reflexology on physiologic parameters and mechanical ventilation weaning time in patients undergoing open-heart surgery: A clinical trial study. Complement Ther Clin Pract. 2015 Aug;21(3):188-92. doi: 10.1016/j.ctcp.2015.07.001. Epub 2015 Jul 6. PMID: 26256138.

25: Çankaya A, Saritaş S. Effect of Classic Foot Massage on Vital Signs, Pain, and Nausea/Vomiting Symptoms After Laparoscopic Cholecystectomy. Surg Laparosc Endosc Percutan Tech. 2018 Dec;28(6):359-365. doi: 10.1097/SLE.0000000000000586. PMID: 30312194.

26: Miralizadeh A, Peyman A, Jamali Soltani N, Ashktorab T. Comparison of the Effect of Foot and Palm Reflexology Massage on Respiratory Distress Syndrome in Premature Infants under Noninvasive Ventilation. Complement Med Res. 2022;29(2):100-108. English. doi: 10.1159/000517982. Epub 2021 Oct 29. PMID: 34818220.

27: Elizabeth AJ, Aruna S, Mercy PJ. Effectiveness of Multi Interventional Package on Selected Parameters of Metabolic Syndrome among Women: A Pilot Study. J Korean Acad Nurs. 2020 Aug;50(4):523-532. doi: 10.4040/jkan.20012. PMID: 32895339.

28: Kito K, Suzuki K. Research on the Effect of the Foot Bath and Foot Massage on Residual Schizophrenia Patients. Arch Psychiatr Nurs. 2016 Jun;30(3):375-81. doi: 10.1016/j.apnu.2016.01.002. Epub 2016 Jan 11. PMID: 27256944.

29: Kim JO, Kim IS. [Effects of aroma self-foot reflexology massage on stress and immune responses and fatigue in middle-aged women in rural areas]. J Korean Acad Nurs. 2012 Oct;42(5):709-18. Korean. doi: 10.4040/jkan.2012.42.5.709. PMID: 23221660.

30: Lee YM, Yeun YR. Effects of Combined Foot Massage and Cognitive Behavioral Therapy on the Stress Response in Middle-Aged Women. J Altern Complement Med. 2017 Jun;23(6):445-450. doi: 10.1089/acm.2016.0421. Epub 2017 May 3. PMID: 28467116.

31: Dong LB, Li YF, Zhang Y, Qiao S. A pilot study of limb stimulation for the treatment of neonatal apnea. Medicine (Baltimore). 2018 Dec;97(49):e12827. doi: 10.1097/MD.0000000000012827. PMID: 30544368; PMCID: PMC6310573.

32: Sudmeier I, Bodner G, Egger I, Mur E, Ulmer H, Herold M. Anderung der nierenduchblutung durch organassoziierte reflexzonentherapie am fuss gemessen mit farbkodierter Doppler-sonographie [Changes of renal blood flow during organ-associated foot reflexology measured by color Doppler sonography]. Forsch Komplementarmed. 1999 Jun;6(3):129-34. German. doi: 10.1159/000021238. PMID: 10460981.

33: Moyle W, Cooke ML, Beattie E, Shum DH, O'Dwyer ST, Barrett S, Sung B. Foot massage and physiological stress in people with dementia: a randomized controlled trial. J Altern Complement Med. 2014 Apr;20(4):305-11. doi: 10.1089/acm.2013.0177. Epub 2013 Sep 18. PMID: 24047244; PMCID: PMC3994911.

34: Koç T, Gözen D. The Effect of Foot Reflexology on Acute Pain in Infants: A Randomized Controlled Trial. Worldviews Evid Based Nurs. 2015 Oct;12(5):289-96. doi: 10.1111/wvn.12099. Epub 2015 Jul 28. PMID: 26220257.

35: Wang HL, Keck JF. Foot and hand massage as an intervention for postoperative pain. Pain Manag Nurs. 2004 Jun;5(2):59-65. doi: 10.1016/j.pmn.2004.01.002. PMID: 15297952.

36: Rollinson K, Jones J, Scott N, Megson IL, Leslie SJ. The acute (immediate) effects of reflexology on arterial compliance in healthy volunteers: A randomised study. Complement Ther Clin Pract. 2016 Feb;22:16-20. doi: 10.1016/j.ctcp.2015.11.001. Epub 2015 Nov 17. PMID: 26850799.

37: Can Çiçek S, Demir Ş, Yılmaz D, Açıkgöz A, Yıldız S, Yis ÖM. The Effect of Aromatherapy on Blood Pressure and Stress Responses by Inhalation and Foot Massage in Patients With Essential Hypertension: Randomized Clinical Trial. Holist Nurs Pract. 2022 Jul-Aug 01;36(4):209-222. doi: 10.1097/HNP.0000000000000526. PMID: 35708557.

38: Lee YM. [Effect of self-foot reflexology massage on depression, stress responses and immune functions of middle aged women]. Taehan Kanho Hakhoe Chi. 2006 Feb;36(1):179-88. Korean. doi: 10.4040/jkan.2006.36.1.179. PMID: 16520577.

39: Ucuzal M, Kanan N. Foot massage: effectiveness on postoperative pain in breast surgery patients. Pain Manag Nurs. 2014 Jun;15(2):458-65. doi: 10.1016/j.pmn.2012.03.001. Epub 2012 Jun 20. PMID: 24882025.

40: Sugiura T, Horiguchi H, Sugahara K, Takeda C, Samejima M, Fujii A, Okita Y. Heart rate and electroencephalogram changes caused by finger acupressure on planta pedis. J Physiol Anthropol. 2007 Mar;26(2):257-9. doi: 10.2114/jpa2.26.257. PMID: 17435375.

41: Park HS, Cho GY. [Effects of foot reflexology on essential hypertension patients]. Taehan Kanho Hakhoe Chi. 2004 Aug;34(5):739-50. Korean. doi: 10.4040/jkan.2004.34.5.739. PMID: 15502439.

42: Chen YS, Lu WA, Clemente FM, Bezerra JP, Kuo CD. Increased Parasympathetic Activity by Foot Reflexology Massage after Repeated Sprint Test in Collegiate Football Players: A Randomised Controlled Trial. Sports (Basel). 2019 Nov 3;7(11):228. doi: 10.3390/sports7110228. PMID: 31684198; PMCID: PMC6915539.

43: Saatsaz S, Rezaei R, Alipour A, Beheshti Z. Massage as adjuvant therapy in the management of post-cesarean pain and anxiety: A randomized clinical trial. Complement Ther Clin Pract. 2016 Aug;24:92-8. doi: 10.1016/j.ctcp.2016.05.014. Epub 2016 May 30. PMID: 27502807.

44: Baljon K, Romli MH, Ismail AH, Khuan L, Chew BH. Effectiveness of Breathing Exercises, Foot Reflexology and Massage (BRM) on Maternal and Newborn Outcomes Among Primigravidae in Saudi Arabia: A Randomized Controlled Trial. Int J Womens Health. 2022 Feb 25;14:279-295. doi: 10.2147/IJWH.S347971. PMID: 35241937;

PMCID: PMC8887672.

45: Holey LA, Dixon J, Selfe J. An exploratory thermographic investigation of the effects of connective tissue massage on autonomic function. J Manipulative Physiol Ther. 2011 Sep;34(7):457-62. doi: 10.1016/j.jmpt.2011.05.012. Epub 2011 Jul 23. PMID: 21875520.

46: Mars M, Maharaj SS, Tufts M. The effect of compressed air massage on skin blood flow and temperature. Cardiovasc J S Afr. 2005 Jul-Aug;16(4):215-9. PMID: 16211126.

47: Hayes J, Cox C. Immediate effects of a five-minute foot massage on patients in critical care. Intensive Crit Care Nurs. 1999 Apr;15(2):77-82. doi: 10.1016/s0964-3397(99)80003-2. PMID: 10595045.

48: Zhang L, Chan P, Liu ZM, Gasbarri M, Lin MT, Chen CW, Hou AL, Chang CY, Chen YC, Chen YS, Chan WP, Leung TK. Evaluation of Reflexology by "BIOCERAMIC Resonance" Operation producing Weak Force Field during Simultaneous Acupoint Stimulation of Urinary Bladder Point on Subject's Ear Resulting in Electric Current Change on Urinary Bladder reflex Point on Subject's Hands, and Related New Research Finding. Acupunct Electrother Res. 2016;41(3-4):207-224. doi: 10.3727/036012917x14831065080096. PMID: 29873994.

49: Moran RW, Gibbons P. Intraexaminer and interexaminer reliability for palpation of the cranial rhythmic impulse at the head and sacrum. J Manipulative Physiol Ther. 2001 Mar-Apr;24(3):183-90. PMID: 11313614.

50: Marsh JP, Turgeon T, Guzman R. Acute limb ischemia following closed reduction of a hip arthroplasty dislocation. Orthopedics. 2010 Oct 11;33(10):768. doi: 10.3928/01477447-20100826-24. PMID: 20954657.

51: KUSZTOS D, KELLER L, FONO J. A SYNKARDIALIS MASSAGE HAT'AS'ANAK RHEOGRAPHI'AS VIZSG'ALATA [RHEOGRAPHIC EXAMINATION OF THE EFFECT OF SYNCARDIAL MASSAGE]. Orv Hetil. 1964 Mar 22;105:550-3. Hungarian. PMID: 14138700.
